# Extraction Optimization, Multi-Dimensional Characterization, and Agricultural Application of Humic Acid from *Protaetia brevitarsis* Frass Derived from Straw Transformation

**DOI:** 10.3390/insects16111084

**Published:** 2025-10-23

**Authors:** Keming Chen, Qi Peng, Ziting Cheng, Lili Geng, Ting Geng, Jie Zhang, Changlong Shu

**Affiliations:** 1 State Key Laboratory for Biology of Plant Diseases and Insect Pests, Institute of Plant Protection, Chinese Academy of Agricultural Sciences, Beijing 100193, China; 2 Langfang Station for Scientific Monitoring and Experiment on Crop Pests, China MOA (Ministry of Agriculture), Langfang 065000, China

**Keywords:** *Protaetia brevitarsis*, convert straw, humic acid, extraction, cherry radish, insect fertilizer

## Abstract

**Simple Summary:**

Agricultural straw is often left unused or burned, leading to resource waste and environmental pollution. Recycling straw with insects offers a sustainable solution. In this study, *Protaetia brevitarsis* larvae converted straw into insect protein and frass, from which humic acid was extracted. The optimized process produced a high-quality humic acid fertilizer that met national safety and quality standards, with abundant nutrients and safe levels of heavy metals. Pot experiments showed that applying the fertilizer significantly promoted cherry radish growth and improved crop quality. This work demonstrates the potential of insect-based recycling to reduce pollution, produce eco-friendly fertilizers, and support sustainable agriculture.

**Abstract:**

Agricultural straw, a massive lignocellulosic by-product, requires high-value utilization strategies, and larvae of *Protaetia brevitarsis* (a resource insect) can convert straw into two valuable products: insect protein and frass rich in humic acid (HA). In this study, we investigated the interactions among multiple parameters affecting HA extraction efficiency and optimized the extraction process. The resulting extract was characterized by elemental analysis to determine nutrient elements, trace elements, and heavy metals; by ^13^C nuclear magnetic resonance (^13^C NMR) spectroscopy to identify the main categories of bioactive molecules; and by pot experiments to evaluate its effects on plant growth and quality. The optimized extraction conditions yielded extracts with a total organic carbon (TOC) concentration of 46.8 g/L, meeting the Chinese standard for water-soluble humic acid fertilizers (NY 1106-2010). Elemental analysis indicated that the extract was rich in trace elements, and heavy metal contents met the limitation requirements of toxic and harmful substances in fertilizers (GB 38400-2019). ^13^C NMR analysis revealed that the extract was enriched in aliphatic and methoxyl carbons, while pot experiments with cherry radish demonstrated that application of the extract at appropriate dosages significantly promoted plant growth and improved crop quality. These findings provide scientific support for circular agriculture and arable land protection, highlighting both their academic significance and broad application prospects.

## 1. Introduction

Agricultural straw, a massive lignocellulosic by-product with annual output exceeding 700 million tons in China, faces critical challenges in high-value utilization. Traditional disposal (open burning, low-efficiency composting) wastes resources and causes environmental issues, making straw conversion into high value products a priority for circular agriculture and carbon neutrality [1].

*Protaetia brevitarsis* (PB) larvae, a saprophagous insect, offer a promising solution by bio-converting straw into dual high value products: insect protein [2,3] and frass rich in humic acid (HA) [4]. Unlike energy intensive chemical methods, larvae decompose straw lignocellulose via gut microbiota endogenous enzyme synergy [5]. The straw derived frass has HA contents far exceeding traditional compost [6], and the larval frass HA was considered as a candidate plant biostimulants. Gao et al. (2023) [7] identified six HA related aromatic compounds from the frass that significantly stimulate plant growth, and all six compounds demonstrated the ability to enhance seed germination, and the piperic acid which was first reported exhibits root growth promotion in plants. Furthermore, the frass extract can be used to develop Eco-friendly pH-responsive hexaconazole nano delivery system for controlling fungal disease and promoting crop growth [8]. These data suggested that further extraction and processing of frass can further enhance its value and promote the high value utilization of straw.

However, the application of frass-derived HA is limited by inefficient extraction. HA is a mixture of macromolecular organic compounds that possess numerous reactive functional groups, such as carboxyl and phenolic hydroxyl groups, which make it readily soluble in NaOH or KOH solutions. Therefore, alkali dissolution methods are commonly used to extract HA from organic materials such as coal [9]. During the extraction process, parameters such as dissolution temperature, dissolution time, alkali concentration, and solid-to-liquid ratio are key factors influencing extraction efficiency. Response Surface Methodology (RSM) has been widely applied to optimize production processes. In this study, RSM was employed to investigate the interactions among multiple parameters and to optimize the HA extraction process.

This study was conducted in response to the growing need for high-value utilization of *P. brevitarsis* larvae frass. It primarily focuses on three aspects: (1) optimization of the extraction process via RSM, (2) physicochemical characterization of the extracted products, and (3) evaluation of the effects of the extracted products on plant growth. The findings of this work are expected to provide technical support for the further development of high-value frass-derived products.

## 2. Materials and Methods

### 2.1. Collection and Processing of Insect Frass Samples

The insect frass used in this experiment was obtained from the population of *P. brevitarsis* reared at the Langfang Pilot Experimental Base of the Institute of Plant Protection, Chinese Academy of Agricultural Sciences. The insects were raised in open plastic boxes with dimensions of 65 cm × 41 cm × 16 cm (length × width × height), fed with fermented Flammulina velutipes culture substrate residue, and maintained under conditions of 25 °C, 70% relative humidity, and total darkness. When most of the feed was consumed, the insect frass and larvae were separated by sieving, and the remaining feed was removed. The sieved insect frass was oven-dried at 75 °C to a constant weight (24–48 h) for subsequent use.

### 2.2. Experimental Design for Response Surface Methodology (RSM) Optimization

#### 2.2.1. RSM Optimization of Factors Affecting Dissolution Rate

To investigate the dissolution properties of insect frass, a three-factor, five-level rotatable central composite design (CCD) was employed in this study, and a quadratic regression model was established. Based on the results of single-factor experiments, liquid-to-solid ratio, KOH concentration, temperature, and time all affected the dissolution rate of insect frass. Three variables, namely X_1_ (liquid-to-solid ratio, g·mL^−1^), X_2_ (extraction temperature, °C), and X_3_ (extraction time, h), were selected as independent variables, with the dissolution rate (%) as the response value. The actual and coded levels of each factor are listed in Table 1, and the experimental design scheme is presented in Table 2.

The experiment consisted of 20 treatments, including 8 full-factorial points, 6 axial points, and 6 center points. The experimental sequence was randomized, and each treatment was repeated three times to minimize systematic errors. The obtained data were used to fit a quadratic regression equation, and analysis of variance (ANOVA) was performed to evaluate the significance and goodness-of-fit of the model. Finally, response surface plots and contour plots were generated to analyze the effects of individual factors and their interactions on the dissolution rate.

The experimental procedure was as follows: According to the liquid-to-solid ratios specified in the table (with a fixed addition of 40 mL KOH solution), insect frass and KOH solution were added to a 150 mL Erlenmeyer flask, and heated with stirring at a constant and moderate speed on a magnetic stirrer for 1 h. Each treatment was repeated three times. After stirring, the dissolved solution was transferred to a 50 mL centrifuge tube, and the residues in the Erlenmeyer flask were rinsed with a small amount of water, which was then added to the same centrifuge tube. Centrifugation was performed at 5000 r/min for 15 min, and the supernatant was discarded; this washing process was repeated five times with ultrapure water. Finally, the obtained precipitate was dried in a constant-temperature oven at 80 °C until its weight remained nearly unchanged, and then weighed to calculate the dissolution rate of insect frass by comparing its weight with the initial weight of the insect frass.

#### 2.2.2. RSM Optimization of Factors Affecting Organic Carbon Content

To optimization of factors to produce a high content extract, a three-factor, three-level Box–Behnken design (BBD) was used to investigate the effects of liquid-to-solid ratio (A, mL/g), KOH concentration (B, mol/L), and extraction time (C, min) on the mass fraction of organic carbon (%) in the extract. The coded levels of factors and the experimental design scheme are shown in Table 3 and Table 4, respectively.

The experimental procedure was as follows: 4 g of insect frass was placed in a 150 mL Erlenmeyer flask, and KOH solution with the corresponding concentration and volume was added according to the liquid-to-solid ratio specified in the table. The mixture was heated and stirred at a constant temperature of 80 °C on a magnetic stirrer at a moderate speed, with the stirring time set according to the experimental design. After the reaction, the solution was transferred to a 50 mL centrifuge tube, and the residues in the Erlenmeyer flask were rinsed with a small amount of ultrapure water, which was then combined with the solution in the centrifuge tube. Centrifugation was conducted at 5000 r/min for 15 min, and the supernatant was collected for the determination of organic carbon content.

### 2.3. Component Analysis (Organic Carbon and Metal Elements)

The organic carbon content was determined according to the standard method described in NY/T 4606-2025 [10].

For the determination of elements including phosphorus (P), potassium (K), copper (Cu), iron (Fe), manganese (Mn), zinc (Zn), boron (B), calcium (Ca), magnesium (Mg), sulfur (S), sodium (Na), as well as heavy metals such as arsenic (As), mercury (Hg), lead (Pb), cadmium (Cd), and chromium (Cr), the procedure followed the Water Industry Standard of the People’s Republic of China SL 394.1-2007 [11], employing inductively coupled plasma atomic emission spectrometry (ICP-AES).

Approximately 0.1–0.5 g of each sample (passed through a 100-mesh sieve) was accurately weighed into polytetrafluoroethylene (PTFE) crucibles. A few drops of ultrapure water were added to moisten the samples, followed by 5 mL of hydrochloric acid (HCl). The mixture was gently heated on an electric hot plate at a low temperature until the volume was reduced to about 2 mL. Then, 10 mL of nitric acid (HNO_3_) was added and heating continued until the solution became nearly viscous. Subsequently, 5 mL of hydrofluoric acid (HF) was added, and the mixture was heated further with frequent shaking to promote silicon removal. Finally, 2 mL of perchloric acid (HClO_4_) was added, and heating was continued until dense white fumes were almost completely expelled.

The inner wall and lid of the crucible were rinsed with 2% nitric acid, and the residue was dissolved while warm. After cooling, the solution was transferred into a 50 mL volumetric flask and diluted to volume with 2% nitric acid. Three reagent blanks were prepared in parallel. The resulting solutions were filtered through 0.22 μm PTFE membranes and analyzed using an inductively coupled plasma atomic emission spectrometer (ICP-AES, Thermo Fisher iCAP 7400 Dual Channel, Thermo Fisher Scientific, Waltham, MA, USA) and an inductively coupled plasma mass spectrometer (ICP-MS, Thermo Fisher iCAP RQ).

The chloride ion content was determined by the silver nitrate titration method, referring to the Agricultural Industry Standard of the People’s Republic of China NY/T 1121.17-2006 [12]. The total nitrogen content was measured using the Kjeldahl method, in compliance with the Agricultural Industry Standard of the People’s Republic of China NY/T 1121.24-2012 [13].

### 2.4. Solid-State ^13^C Nuclear Magnetic Resonance (NMR) Spectroscopy Analysis

The ^13^C cross-polarization magic-angle spinning nuclear magnetic resonance (^13^C CP-MAS NMR) experiments were performed using a JNM-ECZ600R spectrometer (JEOL Ltd., Tokyo, Japan), with the ^13^C resonance frequency set at 150 MHz. Samples were loaded into a 3.2 mm diameter MAS rotor, and data were acquired under magic-angle spinning at 12 kHz. The experimental parameters were as follows: relaxation delay time of 3 s, contact time of 2 ms, and a total of 1200 cumulative scans, resulting in a total acquisition time of approximately 1 h. The line broadening factor was set to 80 Hz.

### 2.5. Preparation of Insect Frass Extract for Plant Experiments

The insect frass, after the aforementioned treatment, was extracted with 0.33 mol·L^−1^ KOH at a concentration of 135 g·L^−1^ and heated at 80 °C for 1 h. After cooling, the mixture was aliquoted and centrifuged at 5000 r·min^−1^ for 15 min. The supernatant was collected, adjusted to neutral pH by dropwise addition of 85% phosphoric acid, and stored at 4 °C. The stock frass extract thus prepared was used for plant experiments in this study. Relevant components of the stock extract were determined, with the total organic carbon content being 25.5 g/L.

### 2.6. Plant Toxicity Assay

The determination of the germination index (GI) was performed following the Agricultural Industry Standard NY 525-2021 [14]. A 10 mL aliquot of the frass extract at the tested concentration was added to 9 mm Petri dishes lined with two layers of qualitative filter paper, and 10 plump, uniformly sized non-coated radish seeds were placed in each dish. Petri dishes containing pure water served as the control. The dishes were covered and incubated in the dark at 27 °C and 60% relative humidity for 48 h. After incubation, the number of germinated seeds was counted, and the primary root length of each seed was measured using a vernier caliper.

The seed germination index (GI) was calculated using the following formula:

GI = [(A1 × A2)/(B1 × B2)] × 100

where

- A1 is the percentage of germinated seeds in the frass extract treatment relative to the total number of seeds (%);
- A2 is the average root length of all germinated seeds in the frass extract treatment (mm);
- B1 is the percentage of germinated seeds in the pure water control relative to the total number of seeds (%);
- B2 is the average root length of all germinated seeds in the pure water control (mm).

The arithmetic mean of parallel determinations was taken as the final result, which was retained to one decimal place. The absolute difference between parallel analysis results did not exceed 5.0%.

### 2.7. Plant Cultivation and Treatment Conditions

Cherry radish (*Raphanus sativus* L.) was used as the material for the cultivation experiment. Uniformly sized seeds were first sterilized with 75% ethanol for 1 min, then soaked in 1% sodium hypochlorite for 5 min, and finally rinsed 3–4 times with sterile distilled water. The treated seeds were soaked in pure water for 6 h, then sown in 11 cm diameter pots containing vermiculite, with 2 seeds evenly sown per pot and 15 pots per treatment group.

On the sowing day, each treatment group was supplied with 2 L of Hoagland nutrient solution with standard formulation. Thereafter, 1 L of 1/2 strength Hoagland nutrient solution was applied every 3 days. PB-F extract, with an original organic carbon content of 25.5 g L^−1^ (prepared as described in Section 2.5), was applied at concentrations of 0.05%, 0.1%, 0.2%, 0.4%, and 0.8% for each treatment group every 6 days together with the nutrient solution, while no PB-F extract was applied to the control group.

Plants were grown in a greenhouse with temperatures maintained at 10–15 °C and a photoperiod of 16 h light/8 h dark. Harvesting and sampling measurements were conducted on day 30. Soluble sugar content was determined using the anthrone colorimetric method; 3 samples were selected from each treatment and assayed using the BC0030 Plant Soluble Sugar Content Assay kit (Solarbio, Beijing, China).

## 3. Results

### 3.1. Basic Characteristics of Insect Frass Samples

#### 3.1.1. Sample Appearance

**Figure 1:**
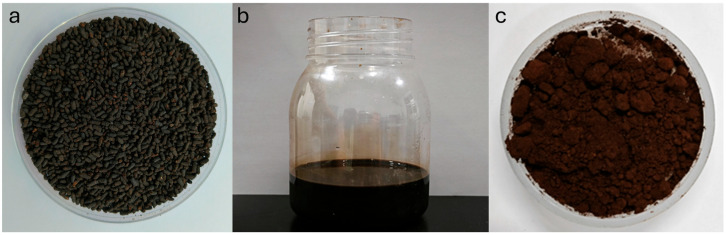
Characterization of PB-frass samples at different stages: (**a**) frass granules, (**b**) frass extract, and (**c**) spray-dried extract.

Freshly collected insect frass was brown and granular, with a high water content and contained impurities. After oven-drying, the insect frass appeared as dark brown granules with a slightly rough surface and uniform particle size (Figure 1a). Following alkaline extraction treatment, a dark brown suspension was obtained; the liquid was slightly turbid, with fine particles uniformly dispersed (Figure 1b). Subsequent to spray-drying treatment using a Holves H-Spray Mini spray dryer (Beijing Holves Biotechnology Co., Ltd., Beijing, China) with an inlet temperature of 120 °C, air flow rate of 40%, and feed rate of 7%, the extract was converted into a light brown fine powder with a fine texture, which was water-soluble (Figure 1c).

#### 3.1.2. Comparison of Component Differences

Based on the elemental content determination results (Table 5), the following changes were observed: First, the total organic carbon (TOC) content in the frass extract was slightly lower than that in the original insect frass. This phenomenon may be attributed to the presence of humin-like substances in the insect frass, which cannot dissolve in alkaline solutions. Second, in terms of nutrient elements: the total nitrogen (TN) content increased slightly, possibly due to the extraction and concentration of water-soluble nitrogen components. The contents of sulfur (S), sodium (Na), and chlorine (Cl) increased significantly, indicating that the compounds of these elements have high water solubility and were concentrated in the extract. In contrast, the contents of phosphorus (P), calcium (Ca), and magnesium (Mg) decreased, suggesting that these elements may exist in insoluble forms or were removed along with the residue. For other trace elements: copper (Cu) and boron (B) contents decreased slightly, while iron (Fe), manganese (Mn), and zinc (Zn) contents decreased significantly—these observations indicate that these elements have low solubility in alkaline extracts.

Most heavy metals (lead [Pb], cadmium [Cd], chromium [Cr]) showed a certain degree of content reduction in the extract, while the contents of arsenic (As) and mercury (Hg) remained basically unchanged. Notably, all heavy metal contents were below the limit requirements specified in the National Standard of the People’s Republic of China GB 38400-2019 [15], demonstrating that the insect frass extract has good safety.

The spray-dried insect frass extract is mainly a concentrate of water-soluble components. It retains most of the organic matter and soluble nutrient elements while reducing some insoluble minerals and heavy metals. This characteristic provides a reference basis for subsequent crop application or further extraction and utilization.

### 3.2. Optimization of Extraction Conditions

#### 3.2.1. Analysis of Factors Related to Insect Frass Dissolution Rate

**Figure 2:**
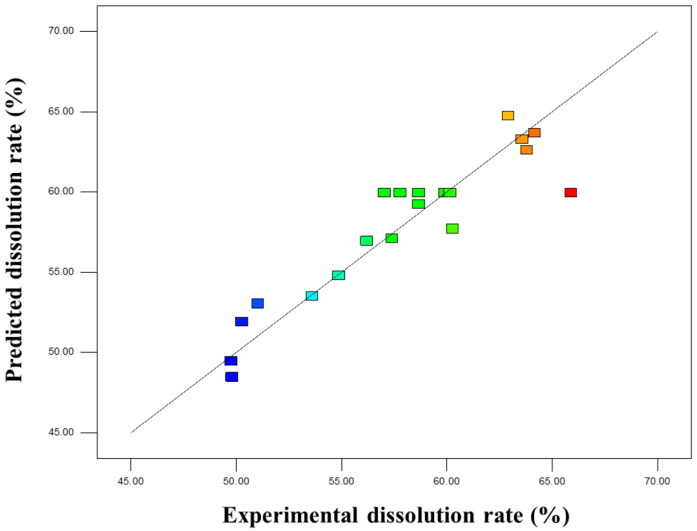
Scatter plot comparing the experimental data and predicted values of insect frass dissolution rate.

Based on the analysis of variance (ANOVA) results in Table 6, the quadratic polynomial model exhibited an F-value of 6.35 (*p* < 0.01), indicating that the model fit was significant and could well reflect the relationship between the insect frass dissolution rate and various factors. The goodness of fit (R^2^) was 0.8511, meaning the model could explain 85.11% of the variation. The predicted values and measured values were distributed near the diagonal line (Figure 2), with small errors, confirming a good fitting effect.

The significance test of each factor (Table 7) showed that the liquid-to-solid ratio (X_1_) and temperature (X_3_) had a highly significant effect on the dissolution rate (*p* < 0.01), while the effect of KOH concentration (X_2_) was relatively minor. The order of the influence of factors was X_3_ (temperature) > X_1_ (liquid-to-solid ratio) > X_2_ (KOH concentration). None of the interaction terms were significant, but the quadratic term X_2_^2^ had a significant effect on the dissolution rate (*p* < 0.05). The regression equation established accordingly is as follows:

$$Y\;(\mathrm{dissolution}\;\mathrm{rate},\;\%)\;=\;59.94+2.64X_{1}+1.39X_{2}+4.11X_{3}-0.0025X_{1}X_{2}+\;0.11X_{1}X_{3}-0.33X_{2}X_{3}-0.251{X_{1}}^{2}-1.62{X_{2}}^{2}-1.26{X_{3}}^{2}$$

**Figure 3:**
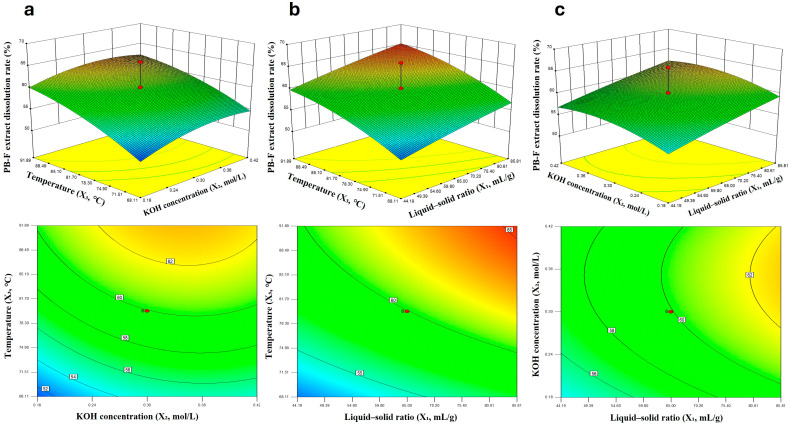
PB-F extract Dissolution Rate Response Surface and Contour Plots (**a**–**c**).

Response surface plots further revealed the interaction relationship between factors (Figure 3). As shown in the figures: (a) The liquid-to-solid ratio had a greater effect on the dissolution rate than the KOH concentration; (b) Temperature had the most significant effect on the dissolution rate, which was stronger than that of the liquid-to-solid ratio; (c) Both temperature and KOH concentration had obvious effects on the dissolution rate, among which temperature exerted a more prominent influence.

Through calculation, the optimal process conditions were determined as follows: liquid-to-solid ratio X_1_ = 183.43 mL/g, KOH concentration X_2_ = 0.33 mol/L, and temperature X_3_ = 102.03 °C, with a predicted dissolution rate of 71.42%. Three replicate experiments were conducted under these conditions, and the average measured dissolution rate was 71.0%, which was highly consistent with the predicted value. This result verified the reliability of the model.

#### 3.2.2. Analysis of Factors Related to Insect Frass Extracts Concentration

In Section 3.2.1, the alkali dissolution conditions for insect frass were investigated using dissolution rate as the indicator. However, in practical production, in addition to improving extraction efficiency, it is also necessary to balance economy and applicability such as reducing transportation costs and meeting the standards for liquid fertilizers. Therefore, the goal of further optimization was shifted to maximizing the concentration of total organic carbon (TOC, g/L) in a single extraction process.

**Figure 4:**
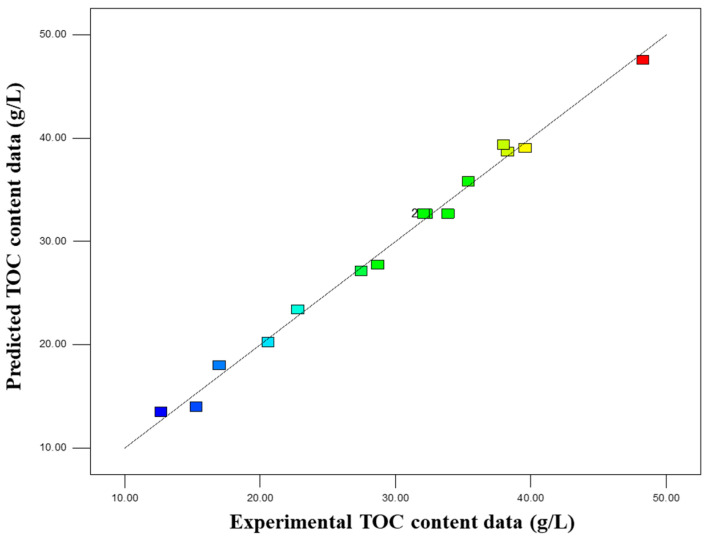
Scatter plot comparing experimental and predicted TOC content of PB-F extract.

**Figure 5:**
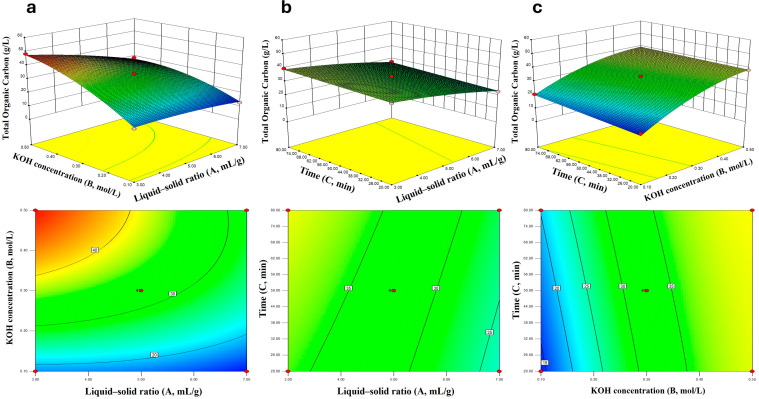
PB-F extract Total Organic Carbon (TOC) Response Surface and Contour Plots (**a**–**c**).

To this end, the Box–Behnken design was employed (with liquid-to-solid ratio, KOH concentration, and time as three factors at three levels), and TOC content was used as the response variable. Analysis of variance (ANOVA) results (Table 8) showed that the model was highly significant (F = 95.60, *p* < 0.0001) with good fitting degree (R^2^ = 0.9931), and the experimental values were consistent with the predicted values (Figure 4). Response surface plots (Figure 5) further revealed that, within the selected test range and gradient, KOH concentration had the most significant effect on TOC, followed by liquid-to-solid ratio, while the effect of time was relatively weak. Among the factors (denoted as A, B, and C, corresponding to liquid-to-solid ratio, KOH concentration, and time, respectively), the linear terms of A, B, and C were all significant (*p* < 0.01); the AB interaction term and the quadratic term B^2^ were also significant. This indicated that there was an interaction between KOH concentration and liquid-to-solid ratio, and KOH concentration had a non-linear relationship with TOC (Table 9). The regression equation was as follows:

$$Y\;(\mathrm{Extracts}\;\mathrm{concentration})\;=\;32.65-6.08A+10.96B+1.74C-3.83AB+0.12AC-\;1.40BC-1.35A^{2}-4.63B^{2}+0.025C^{2}$$

The final optimized conditions were determined as: liquid-to-solid ratio of 3 mL/g, KOH concentration of 0.5 mol/L, and extraction time of 80 min. Under these conditions, the predicted TOC concentration was 47.8 g/L, and the average TOC concentration obtained from verification experiments was 46.8 g/L. The difference between the two values was small, confirming the reliability of the model.

### 3.3. The Solid-State ^13^C NMR Analysis

**Figure 6:**
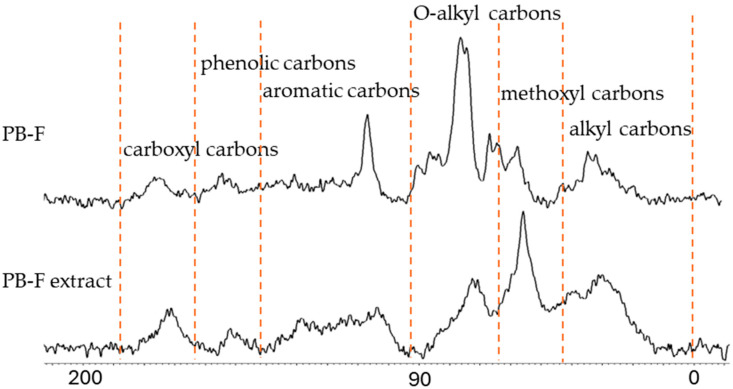
Solid-state ^13^C NMR spectra of PB-F and PB-F extract.

The solid-state ^13^C NMR spectra of PB-F and PB-F extract are presented in Figure 6. Both samples exhibited typical chemical shift signals of organic matter. Specifically, the 0–44 ppm region corresponds to alkyl carbons; 44–64 ppm to methoxyl carbons; 64–93 ppm to O-alkyl carbons; 93–142 ppm to aromatic carbons; 142–162 ppm to phenolic carbons; and 162–188 ppm to carboxyl carbons.

By integrating the peaks across the 0–200 ppm region in Figure 6, the relative abundance of each carbon functional group was calculated (Table 10). In PB-F, aromatic carbon (44.99%) predominates, likely corresponding to lignin-derived carbons and other condensed, recalcitrant structures. In contrast, PB-F extract exhibits a markedly lower aromatic carbon content (11.80%), while alkyl carbon (38.10%) and methoxyl carbon (31.10%) are relatively enriched [6].

These compositional changes indicate that the alkaline extraction process selectively mobilized soluble organic matter into the filtrate. PB-F extract is primarily enriched in aliphatic and methoxyl components, whereas recalcitrant aromatic structures remain largely in the filtered solid residue. Similar trends have been reported for alkali extractions of plant residues and soil organic matter, where soluble aliphatic compounds and lignin-derived methoxyl groups are extracted, while recalcitrant aromatic structures remain in the solid phase [16,17].

From a functional perspective, this suggests that PB-F contains both labile and recalcitrant organic matter, whereas PB-F extract represents the soluble, more bioavailable fraction. The enrichment of aliphatic-rich components in PB-F extract may facilitate faster microbial utilization, while the aromatic-rich residue in PB-F may contribute to long-term carbon stabilization [18].

When PB-F extract and PB-F are applied together, they provide a balance of immediate (from PB-F extract) and long-term (from PB-F) effects, enabling more effective soil amendment.

### 3.4. Results of Plant Toxicity Assay

**Figure 7:**
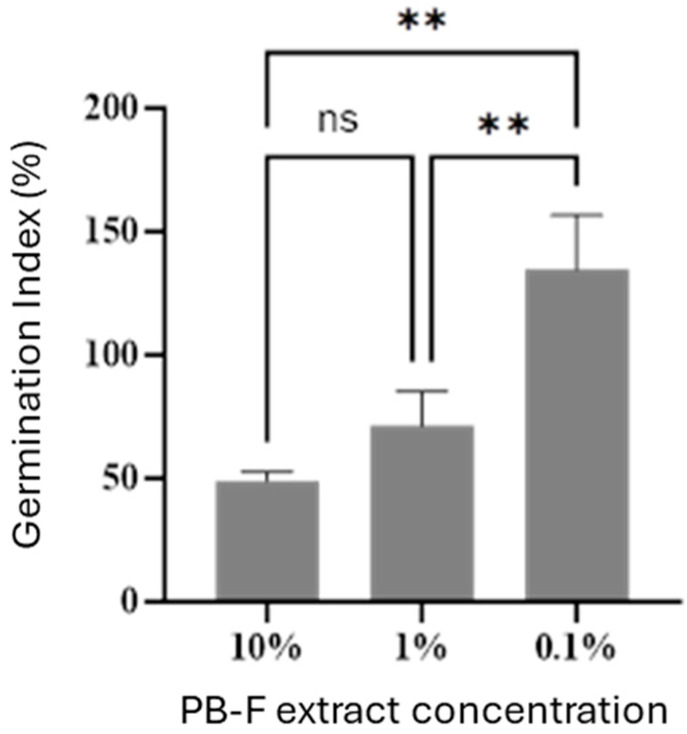
Effect of PB-F extract concentration on the germination index (GI) of radish seeds. The symbols ** represent significance levels of *p* < 0.01.

To evaluate the potential phytotoxicity of the insect frass extract, the germination index (GI) of cherry radish seeds under different concentration treatments was determined. As shown in Figure 7, when the concentration increased to 10%, a significant inhibitory effect was observed, which significantly reduced the seed germination capacity. In contrast, at a concentration of 0.1%, the frass extract exhibited a promotive effect on seed germination, with a GI exceeding 100%—this indicates that low doses of the extract have a good biostimulatory effect. For practical application, a 1000-fold dilution of the extract is required, which is consistent with the recommended dilution practice for liquid organic fertilizers.

### 3.5. Effect of PB-F Extract on Plant Growth

The effect of PB-F extract irrigation on the morphological indices of cherry radishes is shown in Table 11. The root-shoot ratio increased with increasing PB-F extract concentration, reaching the maximum value in the T5 treatment (0.8%), which was 96.3% higher than that of the control (CK). The maximum leaf length and leaf width first increased and then decreased, but the changes were not significant at low PB-F extract concentrations. At higher concentrations, the maximum leaf length in the T4 (0.4%) and T5 (0.8%) treatments decreased by 13.3% and 13.7% compared with CK, respectively, while the leaf width decreased by 15.8% and 14.6%, respectively. In contrast, the fruit diameter increased significantly at high concentrations: compared with CK, the fruit diameter in T4 (0.4%) and T5 (0.8%) increased by 8.2% and 14.6%, respectively. These results indicated that low concentrations of PB-F extract (0.05–0.2%) were beneficial for leaf expansion, whereas high concentrations of PB-F extract (0.4–0.8%) promoted root growth and fruit enlargement while inhibiting leaf growth.

The effect of PB-F extract irrigation on the biomass indices of cherry radishes is presented in Table 12. The total fresh weight increased with increasing PB-F extract concentration, with the highest value observed in the T5 treatment (0.8%)—45.3% higher than that of CK. The fresh weight of the belowground part showed a similar increasing trend: the T5 treatment (0.8%) exhibited a 94.9% increase compared with CK. The fresh weight of the aboveground part first increased and then decreased, with the maximum value (7.53 g) recorded in the T3 treatment (0.2%), which was 26.8% higher than that of CK (5.94 g). In contrast, the aboveground fresh weight in T4 (0.4%) and T5 (0.8%) decreased by 19.7% and 21.0%, respectively. The aboveground dry weight was the highest in the T2 treatment (0.1%), followed by the T3 treatment (0.2%), while the T1 (0.05%), T4 (0.4%), and T5 (0.8%) treatments showed moderate increases relative to CK. The belowground dry weight remained relatively stable across all treatments, ranging from 0.45 to 0.62 g, with no significant differences among groups. These results demonstrated that low concentrations of PB-F extract (0.05–0.2%) facilitated the accumulation of aboveground dry matter, whereas higher concentrations of PB-F extract (0.4–0.8%) mainly promoted the increase in belowground fresh weight, thereby contributing to the elevation of total biomass.

**Figure 8:**
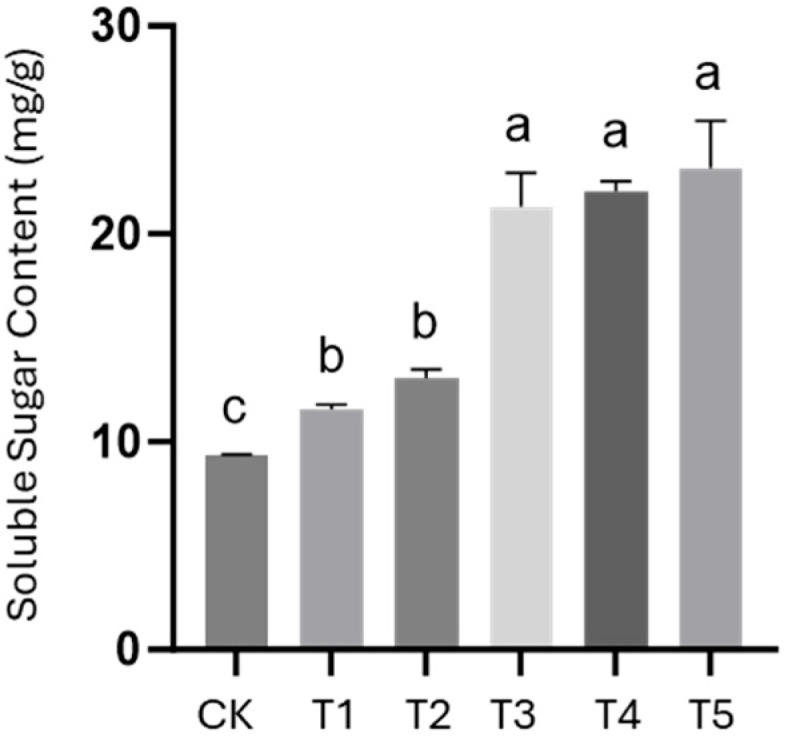
Effects of PB-F extract Treatments (T1–T5: 0.05–0.8%) on Soluble Sugar Content of Cherry Radish. Different lowercase letters above the bars indicate significant differences between treatments (*p* < 0.05).

**Figure 9:**
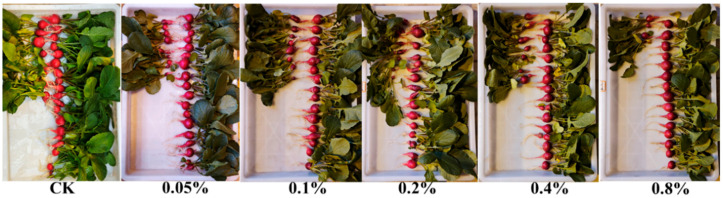
Growth of cherry radish under different PB-F extract treatments: CK = control; T1 = 0.05%; T2 = 0.1%; T3 = 0.2%; T4 = 0.4%; T5 = 0.8%.

Additionally, the effect of different PB-F extract concentrations on the soluble sugar content of cherry radishes was evaluated (Figure 8). Compared with the control (CK), all PB-F extract treatments significantly increased the soluble sugar content; the highest concentration (0.8% PB-F extract) resulted in an increase of up to 147.3% relative to CK. These findings indicated that PB-F extract significantly promoted the accumulation of soluble sugar in cherry radishes, especially at medium and high concentrations—suggesting a positive effect of PB-F extract on root quality. Figure 9 shows photographs of cherry radish groups after harvest.

## 4. Discussion

The global population growth has driven up food demand, intensifying the overexploitation of arable land [19]. This overexploitation leads to land degradation issues such as salinization and organic matter loss, posing a threat to food security [20]. HA can improve soil structure, enhance soil fertility, and boost crop stress resistance, making it a core material for remediating degraded arable land. As an agricultural waste, crop straw can be used as a raw material for artificial HA production [21]. This approach not only addresses straw pollution but also replaces fossil-derived HA, reducing costs and being environmentally friendly. This study was conducted to meet the demands in this field, holding significant implications for environmental sustainability.

Artificial HA is currently a sustainable source of HA, and the mainstream preparation method is microbial transformation, which simulates the natural humification process. However, conventional artificial HA production methods have limitations: biological fermentation (e.g., straw composting) is time-consuming and yields low HA content. In contrast, *P. brevitarsis* plays a crucial role in the ecosystem by transforming plant litter into soil humus [5,22]. When used as a bioreactor, its gut microbiota synergizes with its own metabolism to complete the transformation within a few hours [5]. Moreover, the HA content in the product is higher than that in compost [6]. This approach not only simulates natural humification but also overcomes efficiency bottlenecks and enables straw resource utilization, thereby providing a new pathway for the efficient and environmentally friendly production of biological HA.

In this study, response surface methodology (RSM) was employed to optimize the extraction process. RSM has been widely applied in similar studies (e.g., coal HA extraction) and achieved favorable results [9]. Here, the predicted values of the polynomial model for insect frass dissolution rate showed good consistency with the actual test results. The HA content prepared via the RSM-optimized extraction process reached 47.8 g/L, meeting the industrial standards for soluble organic fertilizers. Further elemental analysis of the spray-dried HA revealed a total NPK content of 163.4 g/kg, and the contents of heavy metals (e.g., Pb, Cd, Cr) were below the limit requirements specified in national standards. Additionally, phytotoxicity tests at the application dose (0.1% concentration of PB-F extract stock solution) showed that the extract exhibited a promotive effect on seed germination, with a germination index (GI) exceeding 100%. Collectively, these data demonstrate that this process is feasible for artificial HA production.

^13^C NMR analysis of the spray-dried extract showed that aliphatic and methoxyl carbons were significantly enriched in the sample after extraction. Compared with industrial natural HA, the artificial HA prepared in this study contains more bioactive molecules. In the pot experiment with cherry tomatoes, frass-derived HA significantly promoted belowground biomass accumulation at an application rate of 0.4–0.8%, which effectively increased crop yield. Furthermore, soluble sugar content analysis indicated that all concentrations of frass-derived HA increased soluble sugar levels; specifically, at 0.8% concentration, the soluble sugar content was 147.3% of that in the control group. This confirms that frass-derived HA can effectively improve crop quality.

In conclusion, this study demonstrates the production of artificial HA via straw transformation by the resource insect *P. brevitarsis*, which provides scientific support for circular agriculture, arable land protection, and the “dual carbon” goals, and thus possesses both academic value and broad application prospects.

## Figures and Tables

**Table 1:** Coded and actual factor levels for the three-factor central composite design (CCD) applied to frass dissolution.

Factor	Code	Factor Levels				
		−1.682	−1.000	0.000	1.000	1.682
Liquid–solid ratio (mL/g)	X_1_	30.000	44.191	65.000	85.809	100.000
KOH concentration (mol/L)	X_2_	0.100	0.181	0.300	0.419	0.500
Temperature (°C)	X_3_	60.000	68.109	80.000	91.891	100.000

**Table 2:** Experimental design of the three-factor central composite design (CCD) for frass dissolution.

Run	Coded Variables			Actual Variables		
	x_1_	x_2_	x_3_	X_1_	X_2_	X_3_
1	−1.0	−1.0	−1.0	44.2	0.2	68.1
2	1.0	−1.0	−1.0	85.8	0.2	68.1
3	−1.0	1.0	−1.0	44.2	0.4	68.1
4	1.0	1.0	−1.0	85.8	0.4	68.1
5	−1.0	−1.0	1.0	44.2	0.2	91.9
6	1.0	−1.0	1.0	85.8	0.2	91.9
7	−1.0	1.0	1.0	44.2	0.4	91.9
8	1.0	1.0	1.0	85.8	0.4	91.9
9	0.0	0.0	0.0	65.0	0.3	80.0
10	0.0	0.0	0.0	65.0	0.3	80.0
11	0.0	0.0	0.0	65.0	0.3	80.0
12	−1.7	0.0	0.0	30.0	0.3	80.0
13	1.7	0.0	0.0	100.0	0.3	80.0
14	0.0	−1.7	0.0	65.0	0.1	80.0
15	0.0	1.7	0.0	65.0	0.5	80.0
16	0.0	0.0	−1.7	65.0	0.3	60.0
17	0.0	0.0	1.7	65.0	0.3	100.0
18	0.0	0.0	0.0	65.0	0.3	80.0
19	0.0	0.0	0.0	65.0	0.3	80.0
20	0.0	0.0	0.0	65.0	0.3	80.0

**Table 3:** BBD Experimental Factors and Levels (Coded Variables).

Factor		Level		
		−1	0	1
Liquid–solid ratio (mL/g)	A	3	5	7
KOH concentration (mol/L)	B	0.1	0.3	0.5
Extraction time (min)	C	20	50	80

**Table 4:** BBD Experimental Design Scheme.

Run	Coded Variables			Actual Variables		
	a	b	c	A	B	C
1	−1	−1	0	3	0.1	50
2	1	−1	0	7	0.1	50
3	−1	1	0	3	0.5	50
4	1	1	0	7	0.5	50
5	−1	0	−1	3	0.3	20
6	1	0	−1	7	0.3	20
7	−1	0	1	3	0.3	80
8	1	0	1	7	0.3	80
9	0	−1	−1	5	0.1	20
10	0	1	−1	5	0.5	20
11	0	−1	1	5	0.1	80
12	0	1	1	5	0.5	80
13	0	0	0	5	0.3	50
14	0	0	0	5	0.3	50
15	0	0	0	5	0.3	50
16	0	0	0	3	0.1	50

**Table 5:** Partial Composition of Raw Frass and Spray-Dried Frass Extract.

	Parameter	Unit	Raw Frass	Spray-Dried Frass Extract
Nutrients	OM	g/kg	572.588	559.459
	TOC	g/kg	332.128	324.512
	TN	g/kg	32.398	35.200
	P	g/kg	9.074	3.994
	K	g/kg	18.189	124.275
	Cupper (Cu)	mg/kg	21.237	20.734
	Iron (Fe)	g/kg	3.772	0.782
	Manganese (Mn)	g/kg	0.246	0.067
	Zinc (Zn)	g/kg	0.111	0.061
	Boron (B)	mg/kg	24.161	10.105
	Calcium (Ca)	g/kg	38.343	12.874
	Magnesium (Mg)	g/kg	7.825	2.643
	Sulfur (S)	g/kg	3.378	5.351
	Sodium (Na)	g/kg	2.596	4.334
	Chloride(Cl)	g/kg	3.018	26.985
Heavy metals	Arsenic (As)	mg/kg	3.012	3.200
	Mercury (Hg)	mg/kg	0.029	0.025
	Lead (Pb)	mg/kg	4.590	2.002
	Cadmium (Cd)	mg/kg	0.390	0.167
	Total Chromium (Cr)	mg/kg	18.717	3.822

**Table 6:** ANOVA of the polynomial model for Protaetia brevitarsis frass (PB-F) extract dissolution rate.

Source of Variation	Sum of Squares	df	Mean Square	F Value	*p* Value	Significance
Model	408.63	9	45.40	6.35	0.0039	***
X_1_—Liquid-solid ratio (mL/g)	95.02	1	95.02	13.30	0.0045	**
X_2_—KOH concentration (mol/L)	26.42	1	26.42	3.70	0.0834	
X_3_—Temperature (°C)	230.82	1	230.82	32.30	0.0002	***
X_1_X_2_	5.0 × 10^−5^	1	5.0 × 10^−5^	7.0 × 10^−6^	0.9979	
X_1_X_3_	0.10	1	0.10	0.014	0.9076	
X_2_X_3_	0.88	1	0.88	0.12	0.7323	
X_1_^2^	0.90	1	0.90	0.13	0.7299	
X_2_^2^	37.74	1	37.74	5.28	0.0444	*
X_3_^2^	22.92	1	22.92	3.21	0.1036	
Lack of fit	21.21	5	4.24	0.42	0.8173	
Residual	71.47	10	7.15			
Pure error	50.26	5	10.05			
Total	480.10	19				
R^2^ = 0.8511, Pre R^2^ = 0.7172						

The symbols *, **, and *** represent significance levels of *p* < 0.05, *p* < 0.01, and *p* < 0.001, respectively.

**Table 7:** Significance test of the polynomial model coefficients for PB-FR dissolution rate.

Factor	Coefficient Estimate	df	Standard Error	95% Confidence Lower	95% Confidence Upper
Intercept	59.94	1	1.09	57.51	62.37
X_1_—Liquid-solid ratio (mL/g)	2.64	1	0.72	1.03	4.25
X_2_—KOH concentration (mol/L)	1.39	1	0.72	−0.22	3.00
X_3_—Temperature (°C)	4.11	1	0.72	2.50	5.72
X_1_X_2_	−2.5 × 10^−3^	1	0.95	−2.1 × 10^−3^	2.1 × 10^−3^
X_1_X_3_	0.11	1	0.95	−1.99	2.22
X_2_X_3_	−0.33	1	0.95	−2.44	1.77
X_1_^2^	−0.25	1	0.70	−1.82	1.32
X_2_^2^	−1.62	1	0.70	−3.19	−0.049
X_3_^2^	−1.26	1	0.70	−2.83	0.31

**Table 8:** ANOVA of the polynomial model for TOC content of PB-FE.

Source of Variation	Sum of Squares	df	Mean Square	F Value	*p* Value	Significance
Model	1440.09	9	160.01	95.60	<0.0001	***
A—Liquid–solid ratio (mL/g)	295.24	1	295.24	176.40	<0.0001	***
B—KOH concentration (mol/L)	961.41	1	961.41	574.41	<0.0001	***
C—Time (min)	24.15	1	24.15	14.43	0.0090	*
AB	58.52	1	58.52	34.96	0.0010	**
AC	0.063	1	0.063	0.037	0.8531	
BC	7.84	1	7.84	4.68	0.0736	
A^2^	7.29	1	7.29	4.36	0.0819	
B^2^	85.56	1	85.56	51.12	0.0004	**
C^2^	2.5 × 10^−3^	1	2.5 × 10^−3^	1.5 × 10^−3^	0.9704	
Lack of fit	7.93	3	2.64	3.76	0.1528	
Residual	10.04	6	1.67			
Pure error	2.11	3	0.70			
Total sum of squares	1450.13	15				
R^2^ = 0.9931, Pre R^2^ = 0.9099						

The symbols *, **, and *** represent significance levels of *p* < 0.05, *p* < 0.01, and *p* < 0.001, respectively.

**Table 9:** Significance test of polynomial model coefficients of TOC content of PB-F extract.

Factor	Coefficient Estimate	df	Standard Error	95% Confidence Lower	95% Confidence Upper
Intercept	32.65	1	0.65	31.07	34.23
A—Liquid–solid ratio (mL/g)	−6.08	1	0.46	−7.19	−4.96
B—KOH concentration (mol/L)	10.96	1	0.46	9.84	12.08
C—Time (min)	1.74	1	0.46	0.62	2.86
AB	−3.83	1	0.65	−5.41	−2.24
AC	0.12	1	0.65	−1.46	1.71
BC	−1.40	1	0.65	−2.98	0.18
A^2^	−1.35	1	0.65	−2.93	0.23
B^2^	−4.63	1	0.65	−6.21	−3.04
C^2^	0.025	1	0.65	−1.56	1.61

**Table 10:** Relative abundance of carbon functional groups in PB-F sand and PB-F extract.

Sample Type	Alkyl Carbon (%)	Methoxyl Carbon (%)	O-alkyl Carbon (%)	Aromatic Carbon (%)	Phenolic Carbon (%)	Carboxyl Carbon (%)
PB-F	12.02	6.73	25.46	44.99	6.87	8.30
PB-F extract	38.10	31.10	12.90	11.80	/	6.90

**Table 11:** Effects of PB-F extract Treatments (T1–T5: 0.05–0.8%) on Morphological Parameters of Cherry Radish.

Treatment	Root-to-Crown Ratio	Maximum Leaf Length (mm)	Maximum Leaf Width (mm)	Fruit Diameter (mm)
CK	1.07 ± 0.48 b	107.07 ± 5.22 a	54.35 ± 4.19 a	21.53 ± 2.96 b
T1 (0.05%)	1.38 ± 0.57 b	108.64 ± 9.94 a	53.47 ± 3.74 a	21.37 ± 3.22 b
T2 (0.1%)	1.32 ± 0.48 b	112.10 ± 10.88 a	57.52 ± 6.60 a	22.47 ± 3.00 ab
T3 (0.2%)	1.06 ± 0.43 b	108.02 ± 9.25 a	55.43 ± 5.71 a	21.64 ± 3.38 b
T4 (0.4%)	1.85 ± 0.51 a	92.79 ± 9.55 b	45.75 ± 5.26 b	23.29 ± 2.69 ab
T5 (0.8%)	2.10 ± 0.62 a	92.39 ± 8.21 b	46.38 ± 5.70 b	24.67 ± 2.36 a

CK = control; T1–T5 = 0.05%, 0.1%, 0.2%, 0.4%, and 0.8% PB-F extract. Values are means ± SD (n = 15). Comparisons were performed between treatments within each column. Different letters indicate significant differences at α = 0.05 according to Duncan’s multiple range test. Root-to-Crown Ratio is dimensionless.

**Table 12:** Effects of PB-F extract Treatments (T1–T5: 0.05–0.8%) on Cherry Radish Biomass.

Treatment	Aboveground Fresh Weight (g)	Belowground Fresh Weight (g)	Total Fresh Weight (g)	Aboveground Dry Weight (g)	Belowground Dry Weight (g)
CK	5.94 ± 0.80 b	5.09 ± 1.53 d	10.67 ± 1.16 c	0.23 ± 0.11 c	0.60 ± 0.21 a
T1 (0.05%)	6.20 ± 1.34 b	5.62 ± 2.47 cd	12.73 ± 2.84 bc	0.24 ± 0.14 bc	0.62 ± 0.22 a
T2 (0.1%)	6.30 ± 1.06 b	7.09 ± 2.08 bc	12.94 ± 3.15 b	0.34 ± 0.14 a	0.59 ± 0.18 a
T3 (0.2%)	7.53 ± 0.87 a	6.30 ± 0.92 bcd	11.68 ± 2.67 bc	0.32 ± 0.09 ab	0.48 ± 0.18 a
T4 (0.4%)	4.77 ± 0.81 c	7.77 ± 1.74 b	13.24 ± 1.94 b	0.28 ± 0.06 abc	0.58 ± 0.11 a
T5 (0.8%)	4.69 ± 0.82 c	9.92 ± 1.94 a	15.50 ± 3.72 a	0.25 ± 0.08 bc	0.45 ± 0.15 a

CK = control; T1–T5 = 0.05%, 0.1%, 0.2%, 0.4%, and 0.8% PB-F extract. Values are means ± SD (n = 15). Comparisons were performed between treatments within each column. Different letters indicate significant differences at α = 0.05 according to Duncan’s multiple range test.

## Data Availability

The original contributions presented in this study are included in the article. Further inquiries can be directed to the corresponding author.

## Abbreviations

The following abbreviations are used in this manuscript:

PB	*Protaetia brevitarsis*
PB-F	*Protaetia brevitarsis* frass
TOC	Total Organic Carbon
RSM	Response Surface Methodology
HA	Humic acid

## References

[1] Ren J., Yu P., Xu X. (2019). Straw utilization in China-status and recommendations. Sustainability.

[2] Kim T.-K., Yong H.I., Kang M.-C., Cha J.Y., Choi Y.-S. (2022). Effect of hydrocolloids on functionality of Protaetia brevitarsis proteins. Food Sci. Biotechnol..

[3] Lee J.-H., Son H., Subramaniyam S., Lim H.-J., Park S., Choi R.-Y., Kim I.-W., Seo M., Kweon H.-Y., Kim Y. (2025). Impact of Edible Insect Polysaccharides on Mouse Gut Microbiota: A Study on White-Spotted Flower Chafer Larva (*Protaetia brevitarsis seulensis*) and Silkworm Pupa (*Bombyx mori*). Foods.

[4] Wei P., Li Y., Lai D., Geng L., Liu C., Zhang J., Shu C., Liu R. (2020). Protaetia brevitarsis larvae can feed on and convert spent mushroom substrate from Auricularia auricula and Lentinula edodes cultivation. Waste Manag..

[5] Wang K., Gao P., Geng L., Liu C., Zhang J., Shu C. (2022). Lignocellulose degradation in Protaetia brevitarsis larvae digestive tract: Refining on a tightly designed microbial fermentation production line. Microbiome.

[6] Li Y., Fu T., Geng L., Shi Y., Chu H., Liu F., Liu C., Song F., Zhang J., Shu C. (2019). Protaetia brevitarsis larvae can efficiently convert herbaceous and ligneous plant residues to humic acids. Waste Manag..

[7] Gao P., Wang K., Qi C., Chen K., Xiang W., Zhang Y., Zhang J., Shu C. (2024). A New Method for Discovering Plant Biostimulants. Plants.

[8] Hu Z., Shu C., Wang M., Yang T., Pei H., Wang T., Sun S., Zhang F. (2024). Eco-friendly pH-responsive iron-doped insect larval frass extract as a hexaconazole nano-delivery system for controlling fungal disease and promoting crop growth. Chem. Eng. J..

[9] Yang Y., Li Y., Zhang Y., Wang M., Wang P., Liu D. (2024). Process Condition Optimization and Structural Feature Analysis of Humic Acid Extraction from Weathered Lignite. ACS Omega.

[10] (2025). Determination of Total Carbon and Organic Matter in Soil—Elemental Analyzer Method.

[11] (2007). Determination of 34 Elements (Pb, Cd, V, P etc.). Inductively Coupled Plasma Atomic Emission Spectroscopy (ICP-AES).

[12] (2006). Soil Testing. Part 17: Method for Determination of Soil Chloride Iron Content.

[13] (2012). Soil Testing. Part 24: Determination of Total Nitrogen in Soil. Automatic Kjeldahl Apparatus Method.

[14] (2021). Organic Fertilizer.

[15] (2019). Limitation Requirements of Toxic and Harmful Substance in Fertilizers. State Administration for Market Regulation.

[16] Kögel-Knabner I. (2002). The macromolecular organic composition of plant and microbial residues as inputs to soil organic matter. Soil Biol. Biochem..

[17] Mao J.-D., Schmidt-Rohr K. (2004). Separation of aromatic-carbon 13C NMR signals from di-oxygenated alkyl bands by a chemical-shift-anisotropy filter. Solid State Nucl. Magn. Reson..

[18] Steinmuller H.E., Chambers L.G. (2019). Characterization of coastal wetland soil organic matter: Implications for wetland submergence. Sci. Total Environ..

[19] Tilman D., Balzer C., Hill J., Befort B.L. (2011). Global food demand and the sustainable intensification of agriculture. Proc. Natl. Acad. Sci. USA.

[20] Liao Y., Yu Z., Kuang L., Jiang Y., Yu C., Li W., Liu M., Guo X., Ye Y. (2025). Analysis of cultivated land degradation in southern China: Diagnostics, drivers, and restoration solutions. Front. Plant Sci..

[21] Wang R., Li D., Deng F., Cao Z., Zheng G. (2024). Production of artificial humic acid from rice straw for fertilizer production and soil improvement. Sci. Total Environ..

[22] Wang K., Li P., Gao Y., Liu C., Wang Q., Yin J., Zhang J., Geng L., Shu C. (2019). De novo genome assembly of the white-spotted flower chafer (Protaetia brevitarsis). Gigascience.

